# Optimization of County-Level Land Resource Allocation through the Improvement of Allocation Efficiency from the Perspective of Sustainable Development

**DOI:** 10.3390/ijerph15122638

**Published:** 2018-11-25

**Authors:** Lijing Tang, Dongyan Wang

**Affiliations:** College of Earth Sciences, Jilin University, Changchun 130061, China; jldx1989@sina.com

**Keywords:** land resource allocation, optimization, allocation efficiency, county level, sustainable development

## Abstract

Land resources provide stable support for economic development in China. However, due to the scarcity of land, the contradiction between agricultural land protection and construction land expansion is prominent. Under such circumstances, optimal allocation of land resources between agricultural and nonagricultural uses is vitally important. In view of the fact that land resources are indispensable inputs for production activities in agricultural and nonagricultural sectors, reducing the efficiency loss of land resource allocation between agricultural and nonagricultural uses is the only way to optimize the process. Counties are the basic administrative units in China, and their improvement of allocation efficiency will help optimize nationwide land resource allocation. This paper constructs models for estimating county-level land resource allocation efficiency from the perspective of sustainable development and searches for countermeasures to improve allocation efficiency. W County is used as an example to demonstrate how to choose these targeted countermeasures. It is concluded that the best way to optimize county-level land resource allocation between agricultural and nonagricultural uses can be found by estimating allocation efficiency from the perspective of sustainable development.

## 1. Introduction

Land resources are indispensable inputs in socioeconomic production activities, and land resource allocation between agricultural and nonagricultural sectors is always an important issue in the economic development of a society. Due to China’s program of economic reforms (also known as “reform and opening-up”), along with China’s rapid economic growth, land resources provide stable support for the rapid development of economies in China. The country’s construction land has expanded rapidly, and the land use structure has also changed tremendously [1]. However, the scarcity of land resource supply leads to the increasingly tense competition for land resources between agricultural and nonagricultural sectors and an increasingly prominent contradiction between agricultural land protection and construction land expansion [2]. Therefore, China faces many challenges in land utilization and management [3,4]. Generally, construction land is an important agent of regional economic development, and especially with the rapid development of China’s urbanization and industrialization, regional development must be supported by a certain scale of construction land. At the same time, agricultural land is important to ensure food security and ecological security. Nowadays, China is facing increasing environmental pressures, and the requirements for agricultural land protection are becoming stricter. However, the construction land reserve is rapidly decreasing, and the contradiction between agricultural land protection and construction land supply is becoming more prominent. The overexpansion of construction land may have negative effects on food security and ecological security, which threatens sustainable development, and excessive protection of agricultural land may restrict the development of regional economies. Therefore, in order to balance survival needs [5,6] and economic development, the only way to alleviate the imbalance between supply and demand of land resources and realize efficient utilization of land is to constantly regulate and optimize regional land resource allocation between agricultural and nonagricultural sectors and to coordinate regional land resource utilization [7,8,9,10].

The research on how to realize optimal allocation of land resources between different uses has received extensive attention. Some studies have suggested that governments should control, adjust, and coordinate land resource allocation, i.e., the final decisions on land resource allocation should be influenced by government policies [4,11,12,13,14,15,16]. However, because these policies are political and cannot meet the requirement of optimization of land resource allocation, optimal land resource allocation between different uses needs positive guidance from governments. Other studies have shown that population [17,18,19,20,21,22], labor [23], economic development [24,25,26,27,28,29,30], technology [16,31,32,33], culture [34], ideology [35], etc. are important factors that influence land resource allocation and should be taken into full consideration when making decisions on optimal allocation of land resources between different uses. Based on different perspectives, scholars have placed various values on land and used different methods to study land resource allocation between different uses [36,37,38,39,40,41]. These methods have the advantage of estimating the ideal allocation scheme of land resources from different perspectives and achieving a best match between different goals, which provide guidance in land resource allocation optimization. In China, studies on land resource allocation between different uses have mainly focused on the macro level (above the municipal level) [42,43,44]. Therefore, some key issues have been neglected in the existing studies.

The first issue is to include the premise of meeting the basic survival needs of human beings into the optimal allocation of land resources. The purpose of resource utilization is to survive, and the basis of human sustainable development is to meet the basic survival needs of human beings, that is, to guarantee the sustainability of human survival. If human beings cannot survive, sustainable development will be meaningless. It must be emphasized that the optimal allocation of resources should reflect the harmony of development and basic survival needs of human beings. Food and ecological environment are the first survival bases of human beings, so from the perspective of sustainable development, land resource allocation must be based on the premise of ensuring ecological security and food security. Therefore, we must first find out the quantity of agricultural land that must be maintained to ensure ecological security and food security. Then, on this basis, we can optimize land resource allocation.

The second issue is the optimal allocation criteria of land resources. One criterion that should be included is the abovementioned requirement for sustainable development. The eternal contradiction between limited resources and human needs causes problems of efficient resource allocation. As the important material basis in human lives, land resources are limited and scarce, but the occupation of land resources for human survival and development is infinite. This triggers problems of efficient land resource allocation. Therefore, the optimal allocation criteria of land resources should include efficient land resource allocation, that is, to improve the efficiency of land resource allocation under the premise of sustainable development because land resource allocation that is bad for the sustainable survival of humans is inherently inefficient. As an important production factor, to achieve the optimal allocation of land resources between agricultural and nonagricultural sectors, we must ensure that the marginal rate of technical substitution (MRTS) of land resources should be equal between agricultural and nonagricultural sectors. At this point, the adjustment of land resources across sectors will not benefit or harm the interests of each sector, and limited land resources can produce the maximum utility value. Therefore, the optimal allocation criteria of land resource should include the equal marginal utility value between different sectors. In reality, however, the unreasonable allocation of land resources between different sectors caused by government intervention and unhealthy competition will lead to waste and inefficiency of land resources. All human activities are aimed at improving living standards, and land resources are put into different sectors to obtain economic benefits so as to improve living standards. However, the improvement of living standards not only means economic development but also the demand for a friendly environment, which requires attention paid to the maximization of ecological value besides economic value in the process of land resource allocation. Agricultural land has special ecological service value, which should be fully considered in the efficiency evaluation of land resource allocation. Only in this way can the equal marginal utility values be regarded as the optimal allocation. Therefore, the optimal allocation criteria of land resource should include full consideration of the ecological service value of agricultural land.

The third issue is the research scale of land resource allocation. Counties are the basic administrative units in China, and county-level optimization of land resource allocation plays a vital role in the efficient utilization of land resources and sustainable development in not only the counties but the country as a whole. Therefore, it is of practical significance to study the allocation of land resources at the county level and the optimal allocation path in China.

Given the above considerations, this paper constructs models to estimate the efficiency of county-level land resource allocation from the perspective of sustainable development. At the same time, this paper searches for countermeasures to optimize land resource allocation by improving allocation efficiency at the county level. A case study of W County is used to demonstrate the specific process of choosing the optimal path to optimize land resource allocation based on estimating allocation efficiency.

The remainder of the paper is organized as follows: Section 2 explains the selection methods of optimal strategy to optimize county-level land resource allocation based on estimating allocation efficiency. Section 3 introduces the data. Section 4 elaborates on the results and discusses related issues. Section 5 concludes the paper.

## 2. Methods

### 2.1. Concept Definition

#### 2.1.1. Agricultural Land and Construction Land

At present, there is no consensus on the definition of agricultural land, but there are some prevailing points. The first point is that agricultural land equals cultivated land in the narrowest definition; for example, agricultural land in Japan specifically refers to the land used for cultivation [45]. The second point is that agricultural land is the land used for cultivation and animal husbandry; for example, the United Nations Food and Agriculture Organization (FAO) stipulates that agricultural land is a combination of cultivated land, grassland, and pasture [46]. The third view is that agricultural land refers to the land directly used for agricultural production; for example, according to the land use classifying system issued by China’s Ministry of Land and Resources [47], agricultural land includes cultivated land, forest land, grassland, aquaculture water, and other agricultural land. The fourth view is that agricultural land refers to the land directly and indirectly used for agricultural production; this view extends the scope of agricultural land and includes the land that is not related to the core value of agricultural land [48], such as rural settlements. Based on the above points and according to the current land classification in China [49], agricultural land in this paper refers to the land with the value of ecological service and used for agricultural production directly, including cultivated land, forest land, garden land, grassland, and water area. We must point out that water area is the sum area of inland rivers, lakes, coastal and inland tidal flats, reservoirs, swags, and ditches. Although inland rivers, lakes, and coastal and inland tidal flats are classified as “unutilized land” in China’s Current Land Use Classification (2017 version), they can be used as fishery land to support agricultural production and are therefore classified as agricultural land in this paper.

The concept of construction land can also be taken in a broad or narrow sense [50]. In the narrow sense, construction land refers to the land that provides operational space for production and other socioeconomic activities of human beings through construction. However, in the broad sense, construction land refers to the land that is used for nonagricultural production without any agricultural purpose. According to the current land classification in China, land resources are divided into three categories: agricultural land, construction land, and unutilized land. Construction land in this paper refers to the land that supports nonagricultural production activities in nonagricultural sectors and does not come under either agricultural land or unutilized land.

#### 2.1.2. The Value of Agricultural Land and Construction Land

The value of land resources is the monetary measurement of its function. As indispensable inputs in production, both agricultural land and construction land have economic production function. Normally, agricultural land bears the regional development of the primary industry, including agriculture, forestry, animal husbandry, and fishery. From this relationship between agricultural land and various parts within the primary industry, cultivated land bears the development of agriculture and animal husbandry, garden land for agriculture, forest land for forestry, and water area for fishery. Agricultural land is also an important part of the ecological environment, and agricultural land not only has economic production function but also ecological service function from the resources–environment standpoint. Therefore, the value of agricultural land includes economic production value and ecological service value. Meanwhile, construction land bears the development of the secondary and tertiary industries. The value of construction land is mainly economic production value.

### 2.2. Theoretical Basis of Optimal Allocation of Land Resources at County Level

In general, land resources of a county are put into production in agricultural and nonagricultural sectors and bear the function of agricultural and nonagricultural land resources, respectively. According to Pareto optimality (also known as Pareto efficiency), from the perspective of the value of land resources, the optimal condition of county-level land resource allocation is that marginal value of land resources are equal between agricultural and nonagricultural uses, i.e., the sum of marginal economic production value and marginal ecological service value of agricultural land is equal to marginal economic production value of construction land.

The Pareto optimality process of land resource allocation at the county level can be described as follows: Suppose the total land resources available in a county is *Q* and the initial state of land resources is all for agricultural land. With the development of the economy, construction needs to occupy a certain proportion of agricultural land and thus the occupied part will be transformed into construction land. However, the transformed quantity should be properly controlled. When the number reaches *Q_optimal_*, the marginal value of land resources are equal between agricultural and nonagricultural uses, and land resource allocation in the county achieves the state of Pareto optimality. In other words, the Pareto optimal allocation quantity of agricultural land is *Q* − *Q_optimal_*, and the Pareto optimal allocation quantity of construction land is *Q_optimal_*. We must point out that the expansion of county-level construction land refers to not only the occupation of agricultural land but also the occupation of unutilized land in China, with the former being the bigger player. Considering the actual situation, there are significant differences in approval management, capital investment, technical operation, etc. between agricultural land occupation and unutilized land occupation. The leading role of unutilized land occupation in land resource allocation is to alleviate the contradiction caused by agricultural land occupied for economic construction. In order to highlight the current grim situation between agricultural land protection and construction land expansion, this study focuses on agricultural land occupied by construction land.

### 2.3. Land Resource Allocation Efficiency in Different Situations and Countermeasures to Improve Allocation Efficiency at the County Level

From the perspective of sustainable development, a certain quantity of agricultural land must be maintained to ensure ecological security and food security in every county, which is the premise of land resource allocation at the county level. Because of the premise, the allocation of land resources between agricultural and nonagricultural sectors may not achieve Pareto optimality state.

**Figure 1:**
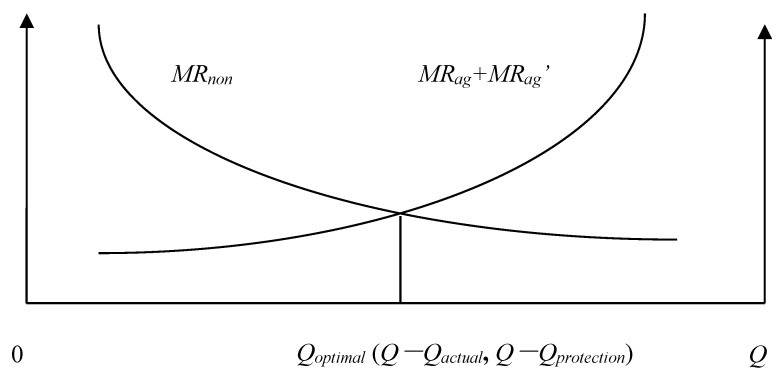
Allocation of county-level land resources under the first situation.

**Figure 2:**
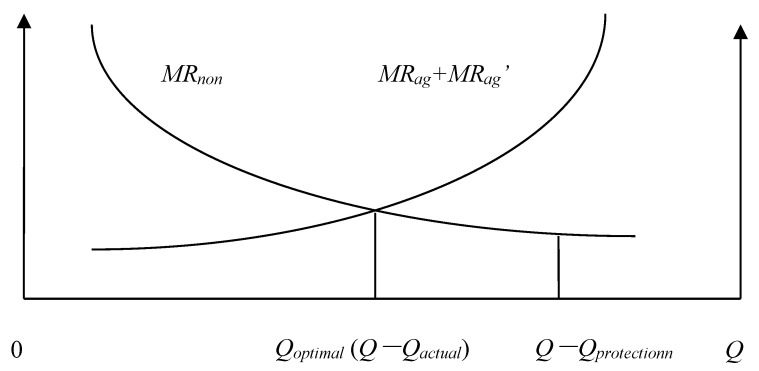
Allocation of county-level land resources under the second situation.

**Figure 3:**
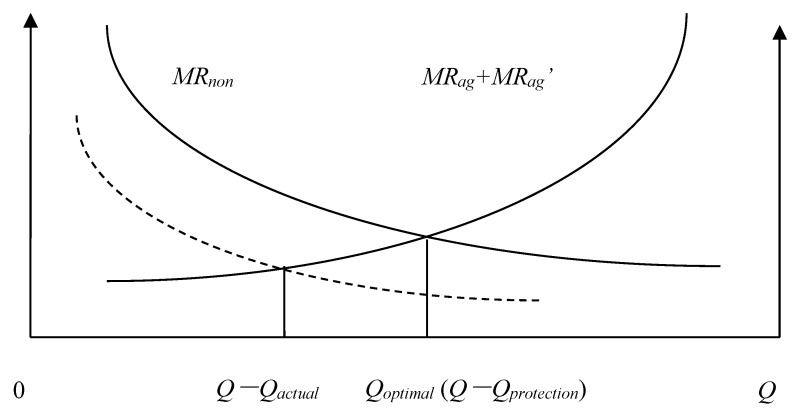
Allocation of county-level land resources under the third situation.

**Figure 4:**
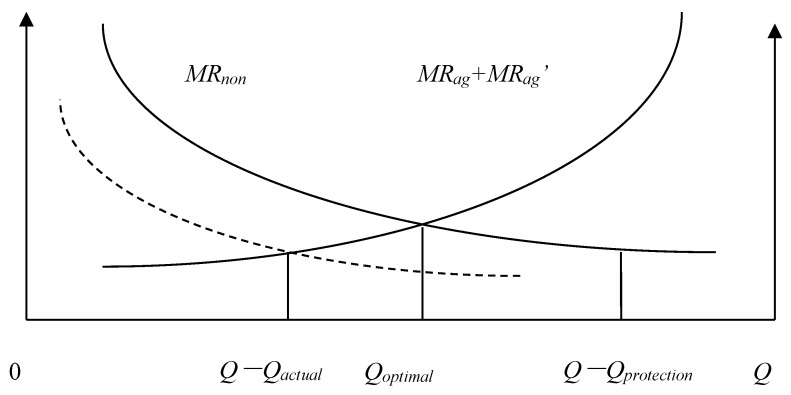
Allocation of county-level land resources under the fourth situation.

**Figure 5:**
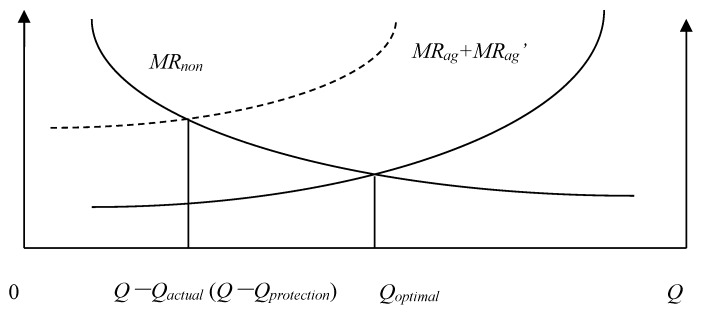
Allocation of county-level land resources under the fifth situation.

**Figure 6:**
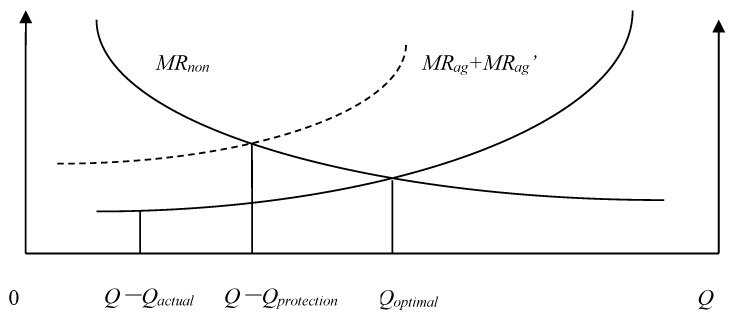
Allocation of county-level land resources under the sixth situation.

**Figure 7:**
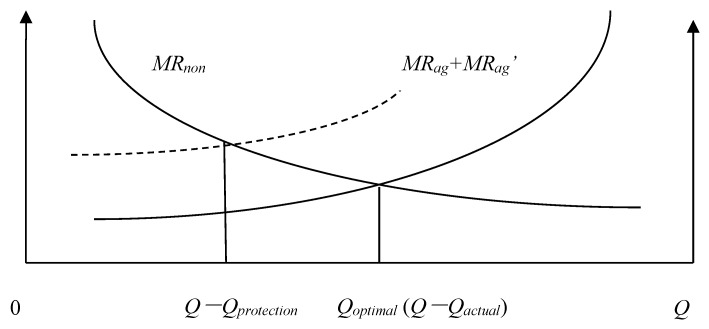
Allocation of county-level land resources under the seventh situation.

**Figure 8:**
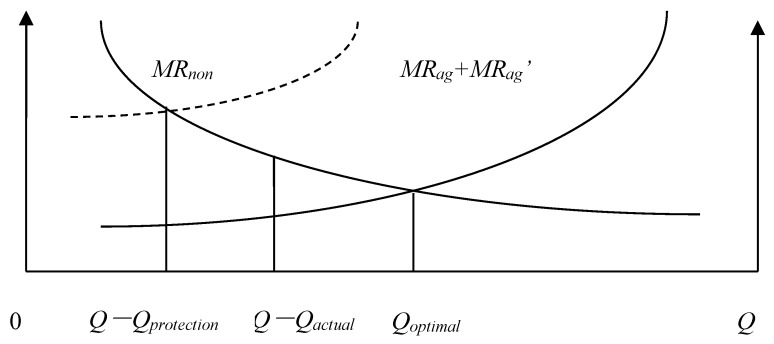
Allocation of county-level land resources under the eighth situation.

We will define the quantity of agricultural land that must be maintained *Q_protection_*, the actual quantity of agricultural land *Q_actual_*, the Pareto optimal allocation quantity of agricultural land *Q* − *Q_optimal_*, the marginal economic production value of agricultural land *MR_ag_*, the marginal ecological service value of agricultural land *MR_ag_*, and the marginal economic production value of construction land *MR_non_*. According to the possible numerical relationships among *Q_protection_*, *Q_actual_*, and *Q* − *Q_optimal_*, the allocation of land resources of a county can be divided into the following eight situations: (1) *Q_actual_* = *Q* − *Q_optimal_* = *Q_protection_*; (Figure 1). (2) *Q_actual_* = *Q* − *Q_optimal_* > *Q_protection_*; (Figure 2). (3) *Q_actual_* > *Q* − *Q_optimal_* = *Q_protection_*; (Figure 3). (4) *Q_actual_* > *Q* − *Q_optimal_* > *Q_protection_*; (Figure 4). (5) *Q_actual_* = *Q_protection_* > *Q* − *Q_optimal_*; (Figure 5). (6) *Q_actual_* > *Q_protection_* > *Q* − *Q_optimal_*; (Figure 6). (7) *Q_protection_* > *Q_actual_* = *Q* − *Q_optimal_*; (Figure 7). (8) *Q_protection_* > *Q_actual_* > *Q* − *Q_optimal_*. (Figure 8). We must point out that when the marginal value of land resources are equal between agricultural and nonagricultural uses, agricultural land will no longer be transformed to nonagricultural uses. Therefore, we will only discuss the situation of *Q_actual_* ≥ *Q* − *Q_optimal_*.

The first two situations are the best state of land resource allocation at the county level, and there is no loss of allocation efficiency in these two situations.

The third and fourth situations belong to excess allocation of agricultural land because the quantity of land resources does not match between agricultural and nonagricultural sectors. This type of allocation efficiency loss can be called efficiency loss of quantity mismatching, represented by *E_n_*. Efficiency loss of quantity mismatching can be reduced by directly adjusting the allocation between agricultural and nonagricultural sectors to optimize land resource allocation. The estimation formula of efficiency loss of quantity mismatching is as follows:

(1) $$E_{n}=\left[Q_{actual}-\left(Q-Q_{optimal}\right)\right]/Q_{actual}$$

For the fifth situation, the allocation of land resources just ensures the quantity of agricultural land that must be maintained, and agricultural land is not allowed to be transformed to construction land. However, due to industrial division, differential rent, and other factors, the marginal value of agricultural land is still less than that of construction land, which means that there is still allocation efficiency loss between agricultural and nonagricultural sectors. The only way to reduce allocation efficiency loss is to improve marginal value of agricultural land in order to narrow the gap of marginal value between agricultural land and construction land. This type of allocation efficiency loss can be called efficiency loss of marginal value gap, represented by *E_v_*. The estimation formula of efficiency loss of marginal value gap is as follows:

(2) $$E_{v}=\left[Q_{protection}-\left(Q-Q_{optimal}\right)\right]/Q_{actual}$$

For the sixth situation, the loss caused by *Q_actual_* > *Q_protection_* is efficiency loss of quantity mismatching, and the loss caused by *Q_protection_* > *Q* − *Q_optimal_* is efficiency loss of marginal value gap. Therefore, the loss of allocation efficiency needs to be reduced by adjusting the allocation between agricultural and nonagricultural sectors and improving the marginal value of agricultural land. This type of allocation efficiency loss can be called efficiency loss of comprehensive gap, represented by *E_t_*. By comparing *E_n_* with *E_v_*, we can choose the more effective way to optimize land resource allocation between agricultural and nonagricultural sectors from adjusting land allocation and improving the marginal value of agricultural land. When *E_n_* is larger, the more effective way to optimize is to adjust land allocation between agricultural and nonagricultural sectors, while when *E_v_* is larger, the more effective way is to improve the marginal value of agricultural land. The estimation formula of efficiency loss of comprehensive gap is as follows:

(3) $$E_{t}=\left[\left(Q_{actual}-Q_{protection}\right)/Q_{actual}\right]+\left\{\left[Q_{protection}-\left(Q-Q_{optimal}\right)\right]/Q_{actual}\right\}$$

The seventh and eighth situations belong to the overexpansion of construction land in which the premise of land resource allocation cannot be guaranteed. This type of allocation efficiency loss can be called efficiency loss of overexpansion, represented by *E_s_*. We must increase the actual allocation quantity of agricultural land to ensure ecological security and food security, while improving the marginal value of agricultural land to optimize the allocation between agricultural and nonagricultural sectors. The estimation formula of efficiency loss of overexpansion is as follows:

(4) $$E_{s}=\left(Q_{protection}-Q_{actual}\right)/Q_{actual}$$

### 2.4. Efficiency Estimation and Optimal Path Selection of Land Resource Allocation at the County Level

Based on the above analysis, efficiency estimation of land resource allocation can follow these steps: (1) calculate the optimal allocation of land resources under Pareto optimality state; (2) calculate the quantity of agricultural land that must be maintained to ensure ecological security and food security; (3) estimate the allocation efficiency of land resources based on actual land resource allocation, the optimal allocation of land resources under Pareto optimality state, and the quantity of agricultural land that must be maintained.

#### 2.4.1. The Optimal Allocation of Land Resources under Pareto Optimality State

This paper applies C-D production function, including the factor of land resources, to simulate the production process of county-level agricultural and nonagricultural sectors. The models are as follows:

(5) $$Y_{ag}=A\times K_{ag}^{a}\times L_{ag}^{b}\times N_{ag}^{c}$$

(6) $$Y_{non}=B\times K_{non}^{e}\times L_{non}^{f}\times N_{non}^{g}$$

where *Y* represents economic production value; *K*, *L*, and *N* represents capital input, labor input, and land input, respectively; *ag* and *non* represent the production process of agricultural and nonagricultural sectors, respectively.

Taking the derivation of land resources variable, the marginal economic production value of agricultural land and construction land can be deduced:

(7) $$MR_{ag}=A\times c\times K_{ag}^{a}\times L_{ag}^{b}\times N_{ag}^{c-1}$$

(8) $$MR_{non}=B\times g\times K_{non}^{e}\times L_{non}^{f}\times N_{non}^{g-1}$$

To further calculate the optimal allocation of land resources between agricultural and nonagricultural sectors under Pareto optimality state, we need to estimate the relationship between the quantity of agricultural land occupied by construction and the marginal value of agricultural land and construction land. According to the supply and demand curves of general merchandise, the supply curve and demand curve for agricultural land occupied by construction can be expressed as Equations (9) and (10), respectively.

(9) $$Q_{D}=h\times P^{-m},\;m>0$$

(10) $$Q_{S}=k\times P^{n},\;n>0$$

where *Q_D_* represents the quantity demanded from agricultural land transformed to construction land; *Q_S_* represents the quantity supplied from agricultural land transformed to construction land; *P* represents the market equilibrium price; *m* and *n* represent demand elasticity and supply elasticity, respectively, where both *m* and *n* are constants. Due to the fact that the period studied was not very long, the demand for land resources in agricultural and nonagricultural sectors was not affected by natural factors in the period studied, so *m* and *n* can be accepted as constants.

In accordance with the pricing formula in microeconomics [51], we know that $MR_{ag}+MR_{ag}{}^{\prime }=P\times \left(1+1/m\right)$, $MR_{non}=P\times \left(1+1/n\right)$. When demand elasticity and supply elasticity are constants, price is positively related with marginal value. Combing these two pricing formulas and deriving them, the marginal value is then related to the quantity of agricultural land occupied by construction. Then, Equations (9) and (10) can be converted to Equations (11) and (12), respectively.

(11) $$\ln Q_{D}=\ln h-m\times \ln MR_{non}+m\ln\left(1+1/n\right)$$

(12) $$\ln Q_{S}=\ln k+n\times \ln\left(MR_{ag}+MR_{ag}{}^{\prime }\right)-n\ln\left(1-1/m\right)$$

To simplify Equations (11) and (12), we let $C_{1}=\ln h+m\ln\left(1+1/n\right)$, $-m=C_{2}$, $C_{3}=\ln k-n\ln\left(1-1/m\right)$, $n=C_{4}$; hence, Equations (11) and (12) can be converted to Equations (13) and (14), respectively.

(13) $$\ln Q_{D}=C_{1}+C_{2}\times \ln MR_{non}$$

(14) $$\ln Q_{S}=C_{3}+C_{4}\times \ln\left(MR_{ag}+MR_{ag}{}^{\prime }\right)$$

where *MR_ag_* and *MR_non_* represent the marginal economic production value of agricultural land and construction land, respectively; *MR_ag_*′ represents the marginal ecological service value of agricultural land; *C*_1_, *C*_2_, *C*_3_, and *C*_4_ are estimated parameters.

After the estimation of *C*_1_ − *C*_4_, letting $MR_{non}=MR_{ag}+MR_{ag}{}^{\prime }$, we can obtain the optimal allocation of agricultural land and construction land during the period studied:

(15) $$Q_{optimal}=\exp\left[\left(C_{1}\times C_{4}-C_{2}\times C_{3}\right)/\left(C_{4}-C_{2}\right)\right]$$

#### 2.4.2. Estimation of the Marginal Ecological Service Value of Agricultural Land

The internationally accepted estimation method used at present for the ecological service value of agricultural land was first proposed by Costanza in 1997 based on the global ecosystem [52]. This estimation method comprehensively considers the supply value of agricultural land (food and raw material production), regulation (gas regulation, purifying environment), support (water conservation, soil conservation, nutrient cycling, and maintenance of biodiversity), and culture (recreation and cultural education). According to Costanza’s method, there is a linear relationship between the total ecological service value of agricultural land and the quantity of agricultural land; in other words, the total ecological service value of agricultural land is equal to the ecological service value of per unit multiplied by the total quantity of agricultural land. The derivation of the total ecological service value of agricultural land (the marginal ecological service value) is equal to the ecological service value of per unit of agricultural land. More intuitively, the marginal ecological service value of agricultural land is equal to the ecological service value provided by per unit of agricultural land. The formula for the marginal ecological service value of agricultural land is as follows:

(16) $$MR_{ag}{}^{\prime }=\sum_{i=1}^{5}\left(S_{i}\times e_{i}\right)/\sum_{i=1}^{5}S_{i}$$

where *S_i_* represents the quantity of cultivated land, forest land, garden land, grassland, and water area and *e_i_* represents the ecological service value of per unit of cultivated land, forest land, garden land, grassland, and water area.

Regarding the ecological service value of cultivated land, forest land, garden land, grassland, and water area and considering the current situation in China, there was a certain deviation in our results from Costanza’s. Therefore this paper adopted the results of Chinese scholar Gaodi Xie, who revised Costanza’s results according to the actual situation in China [53]. Based on the socioeconomic development of W County, we revised the economic value of annual natural grain yield per unit area and calculated the value coefficient of different ecosystem services in the study area. The process of revision is as follows: The average grain yield of W County was 5284.50 kg/hm^2^/a from 2009 to 2017, and the average grain purchase price of W County was 2.36 yuan/kg in 2009. In the absence of labor input, the existing ecological service value was 1/7 of the economic value of grain production provided by cultivated land per unit area, therefore the economic value of natural grain yield of W County was 1781.63 yuan/hm^2^/year.

#### 2.4.3. Estimation of the Quantity of Agricultural Land That Must Be Maintained

Agricultural land includes cultivated land, forest land, garden land, grassland, and water area. The quantity of cultivated land that must be maintained is related to human’s demand for food and is the fundamental guarantee for food security based on the Food Security Act. The formula is as follows:

(17) $$Q_{protection^{\prime }}=\left\{\left[P\times Z\times \left(1+\beta \right)\times r\right]/AP\right\}\times \left(1/F\right)\times \left(1/D\right)$$

where *Q_protection_*_′_ represents the quantity of cultivated land that must be maintained; *P* represents the total population; *Z* represents grain possession per capita; *β* represents grain reserves; *r* represents food self-sufficiency ratio; *AP* represents grain yield per unit area; *F* represents multiple crop index; and *D* represents the proportion of grain planting area to the total planting area of grain crops and cash crops.

The ecological red line is the lifeline for safeguarding national ecological security in China. Its purpose is to establish the most strict ecological protection system and put forward higher regulatory requirements for ecological system, thus comprehensively promoting economic, social, and ecological benefit. Under the leadership of the relevant national departments, every county in China has brought forest land, garden land, grassland, and water area with important ecological service functions into the ecological red line according to its own regional characteristics, so these must be maintained.

Finally, based on the actual land resource allocation, the optimal allocation of land resources under Pareto optimality state, and the quantity of agricultural land that must be maintained to ensure ecological security and food security, we can estimate the efficiency of land resource allocation under the abovementioned eight situations and select the optimal path of land resource allocation optimization of a county.

## 3. Data

### 3.1. Data Description and Data Processing

This paper studied the optimal allocation of land resources under Pareto optimality state using the data of W County between 2009 and 2017. The economic production value of agricultural and nonagricultural sectors was represented by GDP of the primary industry and GDP of the secondary and tertiary industry over the years, respectively. The capital inputs of agricultural and nonagricultural sectors were represented by the total investments in the primary industry and that in the secondary and tertiary industry over the years, respectively. The labor inputs of agricultural and nonagricultural sectors were represented by the number of employees in the primary industry and thatin the secondary and tertiary industries over the years, respectively. The land resource inputs of agricultural and nonagricultural sectors were represented by the total quantity of agricultural land and construction land over the years, respectively.

The estimation of the marginal ecological service value of agricultural land, the marginal ecological service value of cultivated land, garden land, forest land, grassland, and water area of W County, as shown in Table 1, was the actual quantity from 2009 to 2017, and the ecological service value was the actual ecological value from 2009 to 2017 in W County.

Regarding the quantity of agricultural land that must be maintained, the total population and the grain yield per unit area were the actual values in W County at the end of the period studied. The multiple crop index was obtained by dividing the planting area of grain by the area of the cultivated land at the end of the period studied. The proportion of grain planting area to the total planting area of grain crops and cash crops at the end of the period studied was calculated from the crop planting structure. Grain possession per capita, grain reserves, and food self-sufficiency ratio were derived from the food security criteria proposed by the United Nations Food and Agriculture Organization (FAO), which were 400 kg, 18%, and 95%, respectively.

The quantity of agricultural land that must be maintained included the quantity of cultivated land that must be maintained and the forest land, garden land, grassland, and water area in the ecological red line at the end of the period studied.

### 3.2. Data Sources

The Statistical Yearbook of W County (2009–2017) was used to derive data of GDP value; the total investments; the number of employees in the primary, secondary, and tertiary industries; the total population; the grain yield per unit area; and the crop planting structure. The data of the quantity of agricultural land, construction land, cultivated land, forest land, garden land, grassland, and water area, as well as the actual quantity of agricultural land occupied by construction land from 2009 to 2017, were derived from the Bureau of Land Resources of W County. The data on the forest land, garden land, grassland, and water area in the ecological red line were taken from the ecological red line planning of W County.

## 4. Results and Discussion

### 4.1. Estimation Results of Ecological Service Value of Agricultural Land in W County

We obtained the ecological service value of agricultural land from 2009 to 2017 by putting the quantity data of cultivated land, forest land, garden land, grassland, and water area in 2009–2017 and their ecological service value (shown in Table 1) into Equation (16). The results are presented in Table 2.

### 4.2. Estimation Results of C-D Production Function in W County

Based on previous studies, we estimated C-D production function, namely models (5) and (6). We changed the models into logarithmic forms and put the data of GDP, the total investments, the number of employees in the primary industry and agricultural land in 2009–2017 into model (5) and put the data of GDP, the total investments, the number of employees in the secondary and tertiary industry, and construction land in 2009–2017 into model (6) to estimate the relative coefficients. We then used the models’ indexation to obtain C-D production function models in W County.

Next, we put the estimated coefficients as well as the relative data into models (7) and (8) to obtain the marginal economic production value of agricultural land and construction land in 2009–2017, respectively. Then, we put the quantity of agricultural land occupied by construction land, the marginal economic production value of agricultural and construction land, and the marginal ecological service value of agricultural land from 2009 to 2017 into models (13) and (14) to estimate *C*_1_ − *C*_4_. On this basis, we eventually put *C*_1_ − *C*_4_ into model (15), then obtained the optimal allocation of agricultural land and construction land under Pareto optimality state in W County by 2017.

The data were processed by SPSS software, and in order to eliminate the influence of price changes, the GDP value and investment value were converted into the comparable price of 2009. The models were estimated by generalized least-square (GLS). The estimation results are reported in Table 3, Table 4 and Table 5.

By putting *C*_1_ − *C*_4_ into Equation (15), we deduced that the optimal allocation of construction land was 11,531.060 hm^2^ in W County between 2009 and 2017. The total quantity of land resources available in W County was 137,611.300 hm^2^, so the optimal allocation of agricultural land was 126,080.240 hm^2^ in W County.

### 4.3. Estimation Results of the Agricultural Land That Must Be Maintained in W County

In 2017, the total population of W County was 583,000, the grain yield per unit area was 5127.00 kg/hm^2^, the multiple crop index was 1.49, and the proportion of grain crop planting was 0.47. Putting the above data and food safety standards into Equation (17), we could obtain the quantity of cultivated land that must be maintained, i.e., 72,809.276 hm^2^. According to the Red Line Planning for Ecological Protection for the 13th Five-year Plan of W County (2016–2020), the total scale of ecological red line of W County is 41,835.733 hm^2^ in which the forest land is 39,588.61 hm^2^ and the water area is 2247.12 hm^2^. With the total quantity of ecological red line, we can conclude that the agricultural land that must be maintained is 114,645.009 hm^2^.

### 4.4. Efficiency Estimation of Land Resource Allocation and Optimal Path Selection in W County

In 2017, the actual quantity of agricultural land was 136,528.160 hm^2^. Compared with the optimal allocation of agricultural land and the quantity of agricultural land that must be maintained, we can see that the allocation of land resources in W County belongs to the fourth situation in which land resource allocation does not reach the Pareto optimality state due to excessive allocation of agricultural land. In other words, there is efficiency loss of quantity mismatching in W County, and the allocation efficiency loss is 7.653%. Under this circumstance, the optimal path to improve the allocation efficiency between agricultural and nonagricultural sectors in W County is to appropriately reduce the quantity of agricultural land. The excessive allocation of agricultural land in W County is associated with the long-standing development orientation. W County is an underdeveloped agricultural county and has had limited policy influence for a long time because of the lack of geographical advantages. In addition, its natural conditions are especially suitable for agricultural development, so W County has to rely on agricultural production.

### 4.5. Discussion

In reality, the efficiency loss of land resource allocation often exists at the county level in China. For more efficient allocation of land resources and to avoid constant adjustment, we need to find out the more fundamental reasons for efficiency loss and propose targeted countermeasures.

Theoretically, there are two basic means of land resource allocation: the government and the market. In countries with market economy, under the property right of private land ownership, the market mechanism is usually adopted as the basic means of land resource allocation while the government only plays a coordinating and supporting role. However, in China, the government plays a leading role in land resource allocation from the macro background of land resource allocation. The planned mechanism is the main means of land resource allocation, and the allocation of land resources between agricultural and nonagricultural sectors in China is managed through land resource allocation indices by the government at each level. The quantity of land resources allocated to nonagricultural sector is controlled by the total controlling index for construction land, and the quantity of land resources allocated to agricultural sector is controlled by cultivated land protection index and ecological protection index. However, there are some disadvantages in this kind of index control model itself as all indices of each level are decomposed by the superior government and act on the land resource allocation at this level. During the process of index decomposition, there is a gap between the limited knowledge reserve of the index decomposer, incomprehensive information, and the scientific index decomposition demand, which exists in index decomposition at each level. Meanwhile, subjective judgments by the index decomposers is inevitable in the process of index decomposition. Therefore, it is difficult for index decomposers to formulate a perfect index decomposition plan to guide land resource allocation that will result in efficiency loss to land resource allocation at each level, including the county level. At the same time, influenced by the traditional culture of preferring material to service, people tend to pay more attention to and more comprehensively understand the economic value of land resources, while the understanding of the nonmarket value of agricultural land is relatively late and incomprehensive. Under these circumstances, the government decision regarding the planned allocation will also be affected by this traditional culture. Governments often only consider the economic value and ignore the nonmarket value, especially the ecological service value of agricultural land. Therefore, there is an underestimated allocation of agricultural sector based on the underestimation of the ecological value of agricultural land, which will lead to efficiency loss to land resource allocation at each level, including the county level. In addition, with respect to land resource allocation at county level, indices are decomposed from the central to provincial level, municipal level, and finally to each county and act on land resource allocation at county level. During the course where the indices are decomposed from the central to provincial level, municipal level, and county level step by step, except for the abovementioned limitations of index decomposers, the inaccurate estimation on the economic value of land resources and nonmarket value of agricultural land, as well as the inaccuracy in optimal allocation of land resources of governments at all levels caused by the limitations of technical methods, will lead to the deviation of the final decomposed indices at the county level from the optimal allocation quantity under Pareto optimality state. In other words, the stepwise index decomposition progress will lead to efficiency loss in land resource allocation at the county level. Lastly, under the current planned allocation of land resources, indices cannot be freely changed once it is decomposed. Generally speaking, the allocation scale of land resources in nonagricultural sector should not exceed the total controlling index for construction land, and the allocation scale of land resources in agricultural sector should not be less than the protection index of agricultural land. Adverse implementation of indices will affect county governments’ performance. Due to the lack of scientific guidance of political achievements and driven by the maximizing of political interests, the allocation of county-level land resources can only be based on decomposed indices, which results in the deviation of actual allocation quantity from the optimal quantity between agricultural and nonagricultural sectors.

Regarding governments, besides the abovementioned planned allocation, the government management will also cause efficiency loss of land resource allocation at county level. Local governments are not only the administrators of domestic land resources but also the actual power holders of state-owned land ownership, and the overlapping functions induce local governments to maximize economic interests. Although rigidly constrained by indices, the drive for economic goals means there is inevitable deviation of government behaviors, which results in blindly excessive allocation of nonagricultural land resources, unreasonable use and waste of land resources, and efficiency loss of land resources allocation. Resources are always limited or scarce relative to human demand. As a result, there is inevitable competition for resources and conflicts between different interests caused by existing resources in any society. Counties are no exception, and the competition for land between agricultural sector and nonagricultural sector is inevitable for any county. However, the development orientation differs between counties. In counties that are characterized by agricultural development, their agricultural sectors have advantages over nonagricultural sectors in competing for land resources. In contrast, the nonagricultural sectors have advantages over agricultural sectors in competing for land resources in economically strong counties. Without reasonable constraints or regulation to resolve the conflicts between different interests competing for land resources between the two sectors, it will be difficult to realize the optimal allocation of land resources. Finally, the total controlling index for construction land is vitally important for deciding the allocation between agricultural and nonagricultural sectors in China. Because construction land is an important agent of regional economic development, acquiring land for construction means gaining development right. Therefore, this right has always been the focus of competition among governments at all levels, including county governments. In order to avoid the contradictions caused by bias between governments, the superior governments generally follow the principle of “fair right to development” and tend to equally distribute rights for construction land use, thus causing the deviation of given total controlling index for construction land from the optimal allocation of construction land. This means the intervention of municipal governments in the allocation of construction land results in efficiency loss in land resource allocation at the county level.

Based on the abovementioned reasons for efficiency loss of land resource allocation at the county level, we propose some targeted countermeasures.

Due to the limitation of index decomposers, governments at all levels should establish a unified institution to collect and monitor the information—including the information about the economic value of land resources, nonmarket value of agricultural land, and the targets of food security and ecological security under its jurisdiction—so as to guarantee the authenticity and completeness of information provided to index decomposers and finally realize information symmetry at different levels. At the same time, the index decomposers should probe into the scientific quantification of different values of land resources and optimal index decomposition methods with the help of scholars in related fields. With technical improvement and revision of the index scheme, the decomposed indices can be continuously close to the optimal allocation in each region under jurisdiction, thus reducing the deviation caused by stepwise index decomposition to county level land resource allocation. With regard to the fact that governments allocate regional land resources according to the indices driven by political achievements and interests, the municipal governments should try to set up flexible mechanisms for index readjustment to reverse the allocation efficiency loss caused by unreasonable indicators. With regard to governments excessively allocating nonagricultural land resources driven by economic goal and unreasonably allocating land resources between the two sectors based on their own development orientation, the superior governments can adjust the evaluation methods of political achievements and adopt incentive regulation policies. For example, governments can put into practice the principle of Scientific Outlook on Development, change government roles, carry out evaluation of “green GDP”, and include the implementation of food security and ecological security in the government’s performance evaluation. For regions where there is excessive allocation of agricultural land resources, the superior governments and the government itself can formulate policies to attract investment, guide land resources conversion from agricultural sector to nonagricultural sector. Similarly, for regions where there is excessive allocation of nonagricultural land resources, the governments can formulate policies to protect agricultural land and encourage nongovernmental organizations to supervise the protection of agricultural land.

In China, market development is not perfect. As the basic means of land resource allocation, the market does not play a significant role in optimal land resource allocation with the interference of the government. However, governments alone cannot solve some problems related to the optimal allocation of land resources, for example, due to some politics factors. The “fair right to development” principle may hinder the optimal allocation of land resources, that is, the index decomposition of construction land may not be able to scientifically guide the optimal allocation of regional land resources. This phenomenon exists in every level of governments in China, including the county level, so it is sometimes difficult to realize the optimal allocation of county-level land resources. Under this circumstance, the optimization should be realized with the joint effort of the market and the government. Introducing market mechanism to construct exchange platform of indices is a practical way to make up for the loss caused by government intervention. The platform should permit compensated transfer of indices across counties under the guidance of municipal governments; in other words, those counties that suffer allocation efficiency loss due to the restriction of the total controlling index for construction land should be encouraged to purchase index for construction land from the counties whose index is greater than the optimal allocation quantity of construction land. This will be conducive to promoting the optimal allocation of land resources in both county level and higher levels.

This paper can provide references for China’s optimal allocation of land resources at county level. However, the paper still has some limitations that need to be mentioned. The estimation of ecological service value of agricultural land is an important factor to estimate the efficiency of land resource allocation. In this paper, we adopted the most classical and widely used results of Gaodi Xie et al. In the absence of more reliable methods, this method can be used to evaluate ecological service value in China and can be regarded as suitable for China’s ecosystem conditions and economic development. However, there are still many important scientific problems in this method, especially the regional differences of ecological service value. In the future, this method can be used to revise the ecological service value in different regions with different ecological environment characteristics so as to better serve the decision-making of optimal allocation of land resources. In addition, in view of that fact that food security is the ultimate purpose of agricultural land protection and that it has an important function in social stability, this paper represented the social value of agricultural land with the quantity of agricultural land that must be maintained to guarantee food security. Other studies have used social security standard of rural residents and land requisition compensation standard to represent the social value of agricultural land. Still, it is difficult to accurately measure the social value of agricultural land, and the definition of social value of the agricultural land continues to be controversial. For a long time, influenced by the traditional ideas of agricultural land protection, people have often ignored the important social value of construction land, which can absorb labor and safeguard social stability. In the future, studies should evaluate the social service value for agricultural and construction lands based on different regions of different economic development levels and social customs to better serve the decision-making of optimal allocation of land resources.

## 5. Conclusions

Land resources are indispensable inputs in socioeconomic production activities. Due to the scarcity of land, it is important to reduce the efficiency loss in the process of land resource allocation between agricultural and nonagricultural sectors so as to balance survival needs and economic development needs and thus achieve sustainable development. From the perspective of sustainable development, land resource allocation must take ecological security and food security as the premise and be considered from the value of land resources. Counties are the basic administrative units in China and improvements in its efficiency of land resource allocation will help achieve the highest productivity of land resources of the whole nation. Based on the economic theory of Pareto optimality, the premise, and the value of land resources, we constructed models to estimate county-level land resource allocation efficiency between agricultural and nonagricultural sectors and searched for targeted countermeasures to improve allocation efficiency under different situations.

By estimating land resource allocation efficiency in W County and choosing the optimal path for land resource allocation optimization accordingly, we found that it was feasible to optimize the allocation of land resources by improving allocation efficiency. This method provides technical support for land resource allocation optimization at the county level and will help sustainable utilization of land resources and promote sustainable development in China as a whole. We conclude that the best way to optimize land resource allocation between agricultural and nonagricultural sectors can be found by estimating the allocation efficiency from the perspective of sustainable development.

We further analyzed the root causes for efficiency loss of land resource allocation at the county level and proposed a set of policy suggestions, such as guaranteeing information comprehensiveness, taking full consideration of the value of land resource and survival bottom line during the course of index decomposition. Continuously improving index decomposition technology, setting up flexible mechanisms for index readjustment, and introducing market mechanism to construct exchange platform of indices will be helpful for governments to make favorable decisions regarding optimal allocation of land resources at the county level.

## Figures and Tables

**Table 1:** The ecological service value of per unit of cultivated land, forest land, garden land, grassland, and water area in W County (yuan/hm^2^/year).

	Cultivated Land	Forest Land, Garden Land	Grassland	Water Area
Gas regulation	1282.77	7696.64	2672.45	908.63
Climate regulation	1728.18	7251.23	2779.34	3670.16
Water conservation	1371.86	7286.87	2708.08	33,441.20
Soil formation and erosion control	2619.00	7162.15	3990.85	730.47
Waste disposal	2476.47	3064.40	2351.75	26,457.21
Biological diversity	1817.26	8035.15	3331.65	6110.99
Food	1781.63	587.94	766.10	944.26
Raw materials	694.84	5309.26	641.39	623.57
Recreation and culture	302.88	3705.79	1550.02	7910.44

**Table 2:** Results of ecological service value of agricultural land from 2009 to 2017 in W County (yuan/hm^2^).

Year	Ecological Service Value
2009	38,470.53
2010	38,471.26
2011	38,472.63
2012	38,472.93
2013	38,468.80
2014	38,474.73
2015	38,477.74
2016	38,477.55
2017	38,485.10

**Table 3:** Regression results of models (5) and (6) in W County.

**Model (5)**	**A**	**a**	**b**	**c**	**R^2^**
	5.23×10^−163^ *	0.825	−0.006 ***	31.953 *	0.986
(t-statistic)	(−2.359)	(5.545)	(−0.071)	(2.399)	
**Model (6)**	**B**	**e**	**f**	**g**	**R^2^**
	8.66×10^−11^ *	0.250 *	0.053 **	3.482 **	0.996
(t-statistic)	(−2.495)	(3.224)	(2.107)	(3.404)	

Notes: *, **, *** indicate *p* < 0.1, *p* < 0.05, *p* < 0.01, respectively.

**Table 4:** Results of the marginal economic production value of agricultural land and construction land from 2009 to 2017 in W County.

Year	The Marginal Economic Production Value of Agricultural Land (million yuan/hm^2^)	The Marginal Economic Production Value of Construction Land (million yuan/hm^2^)
2009	101.746	333.610
2010	105.887	360.917
2011	103.630	392.958
2012	112.416	414.660
2013	116.008	454.281
2014	116.268	482.906
2015	120.102	493.855
2016	127.852	518.645
2017	131.096	548.326

**Table 5:** Regression results of models (13) and (14) in W County.

**Model (13)**	***C*_1_**	***C*_2_**	**R^2^**
	9.039 ***	0.098 ***	0.946
(t-statistic)	(168.520)	(11.086)	
**Model (14)**	***C*_3_**	***C*_4_**	**R^2^**
	8.770 ***	0.182 ***	0.906
(t-statistic)	(83.560)	(8.233)	

Notes: *** indicate *p* < 0.01.
